# Preparation and Preliminary Evaluation of Neurotensin Radiolabelled with ^68^Ga and ^177^Lu as Potential Theranostic Agent for Colon Cancer

**DOI:** 10.3390/pharmaceutics13040506

**Published:** 2021-04-07

**Authors:** Radu Anton Leonte, Livia Elena Chilug, Radu Șerban, Cosmin Mustăciosu, Alina Raicu, Gina Manda, Dana Niculae

**Affiliations:** 1 Radiopharmaceutical Research Centre, Horia Hulubei National Institute for Physics and Nuclear Engineering, 30 Reactorului Street, Măgurele, 077125 Ilfov, Romania; radu.leonte@nipne.ro (R.A.L.); radu.serban@nipne.ro (R.Ș.); cosmin@nipne.ro (C.M.); alina.raicu@nipne.ro (A.R.); 2 Faculty of Biology, University of Bucharest, 050095 Bucharest, Romania; 3 Victor Babeș National Institute of Pathology, 050096 Bucharest, Romania; gina.manda@gmail.com

**Keywords:** neurotensin, ^68^Ga, ^177^Lu, colon cancer, theranostic, ^68^Ge/^68^Ga generator, uptake, retention, biodistribution

## Abstract

The neurotensin is a tridecapeptide involved in the proliferation of colon cancer, the overexpression of neurotensin receptors occurring at an early stage development of many tumours. Targeting neurotensin receptors by using the same biological active molecule is an effective approach for both imaging quantification and treatment. The present work aimed to demonstrate the ability of radiolabelled neurotensin to specifically target colon cancer cells, and substantiate its usefulness in targeted imaging and radiotherapy, depending on the emission of the coupled radioisotope. Syntheses of ^68^Ga–DOTA–NT and ^177^Lu–DOTA–NT were developed to obtain a level of quality suitable for preclinical use with consistent high synthesis yields. Radiochemical purity meets the pharmaceutical requirements, and it is maintained 4 h for ^68^Ga–DOTA–NT and 48 h for ^177^Lu–DOTA–NT. Extensive in vitro studies were conducted to assess the uptake and retention of ^68^Ga–DOTA–NT, the amount of non-specific binding of neurotensin and the effect of ^177^Lu–DOTA–NT on HT–29 cells. In vivo biodistribution of ^68^Ga–DOTA–NT revealed significant uptake at the tumour site, along with fast clearance evidenced by decreasing activity in kidneys and blood after 60 min p.i. ^177^Lu–DOTA–NT exhibited similar uptake in the tumour, but also a significant uptake at 14 days p.i. in the bone marrow was reported. These results successfully demonstrated the potential of neurotensin to deliver imaging/therapeutic ^68^Ga/^177^Lu radioisotopes pair, but also the need for further evaluation of the possible radiotoxicity effects on the liver, kidneys or bone marrow.

## 1. Introduction

In recent years, an increased contribution has been brought to the development of drugs capable of both imaging specific targets (tumours) and treating the malignant tissue via local irradiation with minimal detriment to the body. Research in the field of theranostic uses certain biological active molecules to which different radioisotopes can be bound for either diagnosis or treatment. This approach aims to find the molecule with high specificity for a tumour cell type, and then properly attach to it an imaging or a treatment radioisotope, according to its use. One molecule of this kind is the neurotensin (NT) for which it has been previously demonstrated to have the potential to target tumours such as: pancreatic cancer [1,2,3], colorectal cancer [4,5,6], lung cancer [6], prostate cancer [7,8] or breast cancer [9]. Neurotensin is a tridecapeptide (pGlu-Leu-Tyr-Glu-Asn-Lys-Pro-Arg-Arg-Pro-Tyr-Ile-Leu) that acts as a neurotransmitter or neuromodulator in the central nervous system (CNS). It is a regulating factor in normal and cancerous tissue growth and also contributes to fat storage, obesity and metabolic disorders [10]. Neurotensin exerts its effects primarily through neurotensin receptor subtype-1 (NTRS1) and with lower affinity through neurotensin receptor subtype-2 (NTRS2), both G-protein–coupled receptors (GPCR), and later-discovered neurotensin receptor subtype-3 (NTRS3), a single transmembrane domain sorting receptor. These findings suggest that the NT signalling pathway may provide a useful target for the development of diagnostic agents and antitumor drugs.

Although previous studies showed that neurotensin has a low stability in blood [11], this work aimed to assess the reliability of neurotensin radiolabelling process, according to pharmaceutical requirements and the potential use for imaging and therapy, depending on the characteristics of the bound radioisotope. The foremost objective of this study was to evaluate the behaviour of the radiolabelled neurotensin to determine to which extent it can be used in natural conditions, and to draw a baseline for further biological studies, e.g., using inhibited proteolytic enzymes and/or pharmacokinetics modifiers.

**Figure 1:**
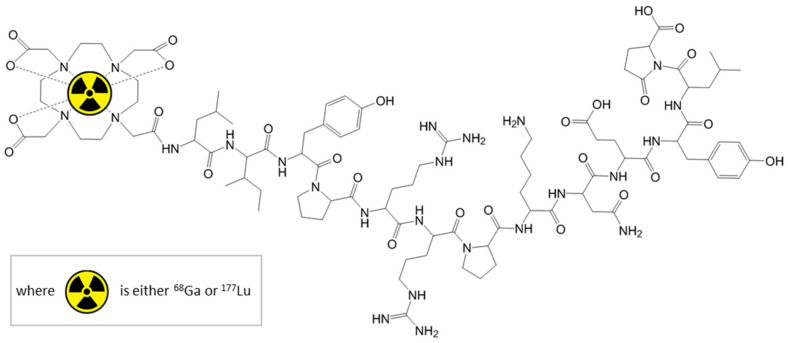
Radiolabelled DOTA-Neurotensin.

The emerging interest in theranostic applications led to the development of peptides coupled with imaging/therapeutic radioisotopes pairs that quickly localize to cancer cells, delivering locally the diagnostic/therapeutic effect, respectively. A major and important choice is selecting the proper radionuclides for the intended use, as there are no “ideal” medical radioisotopes, and then choosing a pair of them for theranostic applications. Such an example of radioisotopes pair is ^68^Ga—used as imaging agent—and its therapeutic counterpart—^177^Lu [12]. Macrocyclic chelating agents, like DOTA can form complexes with radiometallic cations and can bind covalently to biomacromolecules. The ability to complex various cations offers the possibility to use the same biological active molecule both for diagnostic and therapy, depending on the complexed radioisotope (Figure 1).

Gallium has no known natural role in biology, but ^68^Ga has a short half-life, compatible with the pharmacological distribution time of certain biomolecules (e.g., peptides, proteins and oligonucleotides). Furthermore, ^68^Ga and ^177^Lu are two radioisotopes which can be chelated with good results to a variety of biomolecules; this allowing to approach more diseases than e.g., ^99m^Tc. Therefore, ^68^Ga has gained increasing importance in clinical research in the last 25 years [13] as a PET imaging agent, exhibiting more suitable characteristics than other PET radioisotopes in terms of higher detection resolution and sensitivity (T_1/2_ = 67.83 min, E_β+_ = 1.899 MeV) [8,14], a lower or comparative effective dose to that of ^18^F or ^99m^Tc (2.3 mSv for [^68^Ga]Ga-DOTA-TOC per PET scan, compared to 5.6 mSv for [^18^F]FDG [15]) and fast data acquisition. It decays by β^+^ emission (88.88%) and electronic capture (11.11%) to stable ^68^Zn [14].

Currently, the most common method for obtaining ^68^Ga is via ^68^Ge/^68^Ga generator (^68^Ge decays 100% by electronic capture to ^68^Ga), with the alternative of proton irradiation at medium energy cyclotrons via ^68^Zn(p,n)^68^Ga nuclear reaction [16]. Both methods have specific advantages (generator: no gallium impurities produced, virtually carrier-free compounds, minimal radioprotection equipment, ease of operation; cyclotron: much more activity available, no dead-time between batches), but also disadvantages (generator: maximum available activity is limited and decreasing with the age of the generator, limited elutions per day, limited number of patients per batch; cyclotron: radionuclidic impurities produced during irradiation, need of laborious purification methods, higher operation costs, dedicated vault, more expensive radiological protection). Considering these aspects and the needs for our studies, we chose the generator method of ^68^Ga production.

The most widely used ^68^Ga–based imaging/diagnostic agents are [^68^Ga]Ga-DOTA-Somatostatin analogues like TOC, TATE or NOC for patients affected by neuroendocrine tumours (NET), and prostate specific membrane antigen [^68^Ga]^68^Ga-PSMA for prostate tumours [17,18].

^177^Lu is used for therapy due to low energy β^-^ emission (E_β_- = 498 keV), but also provides imaging information due to its low energy gamma photons (E_γ_ = 208.36 keV (10%) and 113 keV (6.2%)) [19,20]. It is obtained in reactor-based production facilities, and its half-life (6.65 days) is sufficiently long for delivery to clinical sites. Major clinical applications of ^177^Lu include therapy with [^177^Lu]Lu-DOTA-TATE for metastatic neuroendocrine tumours [21], [^177^Lu]Lu-PSMA for prostate cancer and [^177^Lu]Lu-DOTA-Rituximab for non-Hodgkin lymphoma [22].

Due to the short half-life of ^68^Ga, it is essential to establish standardized processes, with constant reproducibility, in order to shorten the radiolabelling time and to obtain radiolabelled peptide solution of pharmaceutical grade purity, suitable for injection. The automation of the process is also important, as long as this allows shortening of the overall process time, along with the reduction of radioactive dose to which the operators are exposed.

The present work describes our fast method for labelling the NT peptide with ^68^Ga and ^177^Lu respectively, and assesses the binding kinetics, as well as the biodistribution of ^68^Ga-DOTA-NT and ^177^Lu-DOTA-NT to organs for the applications of NT as a theranostic agent.

## 2. Materials and Methods

All reagents and solvents were purchased from commercial suppliers and used with no other purification. The peptide employed in all experiments (DOTA-NT) was provided already conjugated with DOTA chelator by piChem GmbH (Raaba-Grambach, Austria). Ultrapure water used in synthesis and for preparation of solutions was freshly prepared on-site with Millipore Milli-Q Direct 8 system (Millipore S.A.S., Molsheim, France).

Nomenclature statement: ^68^Ga and ^177^Lu employed in the studies presented in this work comply with the definitions of no-carrier-added and carrier-free compounds according to IUPAC nomenclature (Gold Book [23,24] and Orange Book [25]), and Radiochemistry Society (Richland, WA, USA) [26]. Therefore, the radiolabelled compounds produced and used in our studies are referred, and their chemical formulae are written according to the rules for mentioned definitions.

### 2.1. ^68^Ga-DOTA-NT Synthesis

The preparation of ^68^Ga-DOTA-NT, consisting of radiolabelling with ^68^Ga, purification and formulation, was carried out on the automated synthesis module Galigand GAL-102 (Veenstra Instruments, Joure, The Netherlands).

In order to efficiently use the available activity, the syntheses were carried out just before the biological assays, and the elution was integrated as the first step of the synthesis sequence.

^68^Ga was freshly extracted for each batch from organic–column type ^68^Ge/^68^Ga generator (ITG Isotope Technologies Garching GmbH, Garching, Germany), which is coupled to synthesis module.

The buffer employed in all experiments is 1 M ammonium acetate solution prepared on-site from ammonium acetate salt (Carl Roth GmbH, Germany). For each batch, ^68^Ga was eluted according to manufacturer’s protocol with 4 mL 0.05 M HCl prepared on-site by diluting ultrapure-grade 30% HCl (Merck KGaA, Darmstadt, Germany).

The proper fraction of eluate, consisting of 2 mL of ^68^GaCl_3_ in 0.05 M HCl (pH = 1–1.3), which concentrates most of the activity eluted from the ^68^Ge/^68^Ga generator, was sent immediately in the reaction vessel, already heated to the labelling temperature (95 °C).

Aliquots of 50 µL containing 24 nmol of DOTA-NT dissolved in buffer solution were used for each batch. The amount of buffer required to adjust the pH of the reaction mixture in the range of 4.5–4.8 was previously mixed with the peptide aliquot, in order to protect the biomolecule and to avoid a possible degradation when added to the highly acidic eluate. The peptide-buffer mixture was sent to the labelling vial immediately after the transfer of the ^68^Ga eluate and left to react for 5 min at 95 °C.

After the labelling step, a small amount of ultrapure water (300 µL) was added in the labelling vial, and then the product was passed through a previously conditioned separation cartridge C18 Strata-X SPE (Phenomenex, Torrance, CA, USA). The SPE cartridge retains only the labelled peptide, while the free ^68^Ga impurities are evacuated as reaction waste. The complete removal of impurities was carried out by rinsing the cartridge with 1 mL mixture made of 950 µL ultrapure water and 50 µL ethanol (Chimreactiv S.R.L., Bucharest, Romania). After purification step, the peptide was recovered into the evaporation vial by passing 1 mL of ethanol through the SPE cartridge. Further, in the evaporation vial already heated at 95 °C, the ethanol is evaporated using dry heat and vacuum to hasten the process. The labelled peptide was reconstituted in 1 mL physiological saline solution (0.9% NaCl, B. Braun Melsungen AG, Melsungen, Germany). At the end of the synthesis, ^68^Ga-DOTA-NT was filtered through 0.22 µm membrane filter Millipore (Merck KGaA, Darmstadt, Germany) for sterilization. The pH was measured using pH strips (pH-Fix 2.0–9.0 and pH-Fix 3.6–6.1) from Macherey–Nagel GmbH & Co. (Düren, Germany).

### 2.2. ^177^Lu-DOTA-NT Synthesis

^177^Lu was acquired in form of ^177^LuCl_3_ from National Centre for Nuclear Research—Radioisotope Centre POLATOM (Otwock, Poland). The labelling of neurotensin with ^177^Lu was carried out manually in a preheated reaction vial at 100 °C. 370 MBq of ^177^LuCl_3_ solution was mixed with 1 M ammonium acetate solution in order to adjust the pH value to 5.5, and then 24 nmol of DOTA-NT was added. The mixture was left to react for 10 min under continuous stirring at 100 °C. Then, the heating was turned off and the mixture was left to cool down under stirring for 10 min. The pH was finally adjusted to 6.5–6.8 with buffer. The final formulation of ^177^Lu-DOTA-NT has been done with 1 mL of 0.9% NaCl solution, followed by filtration through a 0.22 μm membrane filter.

### 2.3. Radiochemical Purity and Stability

The radiochemical purity (RCP) of the radiolabelled DOTA-Neurotensin conjugates was assessed by radio-HPLC, using a Shimadzu Prominence 20 A series system (Shimadzu Corporation, Kyoto, Japan) equipped with LB513 BGO γ–detector (Berthold Technologies GmbH & Co.KG, Bad Wildbad, Germany). The stationary phase used was a C_18_ column—Nucleosil^®^ 5µm, 250 mm × 4.6 mm, (Supelco Inc., Bellefonte, PA, USA). The mobile phase was composed of water and acetonitrile, both acidified with 0.1% TFA, at 1 mL/min flow rate. The following gradient was used: 0–2 min 5% B, 2–32 min 5–65% B, 32–35 min 65–5% B, 35–40 min 5% B (where A = water with 0.1% TFA, and B = acetonitrile with 0.1% TFA).

The stability of ^68^Ga-DOTA-NT was assessed by radio-HPLC for 4 h after the end of the labelling and purification process (EOS). 5 samples taken from the same vial were injected at different moments after the end of the synthesis, as follows: immediately, after 1, 2, 3 and 4 h respectively, and evaluated in terms of radiochemical purity. The radiochemical stability of ^177^Lu-DOTA-NT was checked up to 48 h from the end of preparation. At the end of the evaluation period, the radiochemical purity should be higher than 95%.

### 2.4. Cell Culture

The human colon cancer cell line HT-29 (ATCC, Manassas, VA, USA) was incubated in Dulbecco’s Modified Eagle’s Medium (DMEM) (Biological Industries, Beit-Haemek, Israel), at 37 °C in controlled atmosphere containing 5% CO_2_.

For binding assay, Petri dishes (Nunclon^TM^ Delta Surface 150350, ThermoFisher Scientific, Roskilde, Denmark) were prepared with cell culture 24 h before experiments, in order to allow the cells to firmly attach to the dish surface. 4 × 10^5^ cells were seeded in a well-defined area of the dish, while keeping the Petri dish in a tilted position. Once the cells have attached, the remaining cell solution was removed, and 2 mL of DMEM was added. Each dish was inspected under a microscope to check that all cells have attached in the designated area.

### 2.5. In Vitro Uptake and Retention Assessment of ^68^Ga-DOTA-NT

**Figure 2:**
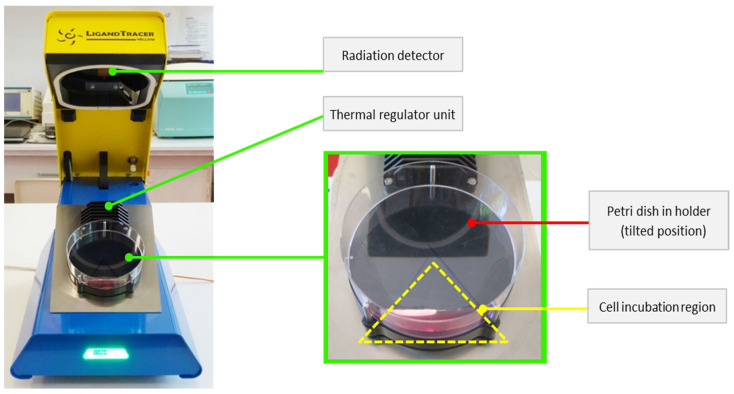
The LigandTracer Yellow instrument with cell culture and DMEM on Petri dish.

The LigandTracer Yellow system (Ridgeview Instruments AB, Uppsala, Sweden) was used to evaluate the amount of tracer that binds to specific receptors expressed on the surface of the tumour cell membrane. This instrument uses the rotating radioimmunoassay (RIA) method to evaluate real-time dynamics of tracer binding to tumour cells during analysis. Every 5.6 min, the system records the activity in 12 points (6 points corresponding to the upper position of the tilted dish and 6 points for the base position—see Figure 2 [27]). The recorded result represents the difference in detected signal between each corresponding point from the two areas [28,29].

To perform this analysis, the background was measured (eliminating any radioactive source around the device for baseline acquisition). 0.6 nmol of ^68^Ga-DOTA-NT with 10 MBq was added over cells in Petri dish and recording was started to obtain the tracer uptake profile. After reaching the steady state, the radioactive medium was removed and the cells were washed with 2 mL of fresh DMEM (1 mL at a time) to remove additional activity (labelled peptide left unbound) from the Petri dish. After this washing step, 2 mL of medium was added over the cells to ensure an optimal survival environment during the next step of the analysis. Cell retention is obtained by restarting the measurement, the retention curve is recorded until a plateau is reached due to the equilibrium state. The same procedure was used in the case of ^177^Lu, but using 0.6 nmol of ^177^Lu-DOTA-NT with 25 MBq activity.

### 2.6. In Vitro Receptor Binding of DOTA-NT and Competition Assay

Neurotensin exhibits three specific receptors with increased affinity against NTRS1 compared to NTRS2 and NTRS3. In order to evaluate the specific binding due to the exclusive interaction of the peptide with its receptors, a competition assay was performed. This assay in which all NT receptors are blocked before incubating the labelled peptide is intended to highlight non-specific binding of NT to the cells. The difference between the results obtained by incubating the non-blocked peptide (total binding) and those obtained with peptide after blocking all receptors (non-specific binding) represents specific binding. For this assessment, three different NT receptors antagonists were used, each having a preferential increased affinity for one of the receptors, allowing the blocking of all NTRs at the same time:

NTRC 824, *N*-[2-[5-[[(4-Methylphenyl) sulfonyl] amino]-3-(trifluoro acetyl)-1H-indol-1-yl] acetyl]-L-leucine is a non-peptide NTSR2 antagonist that exhibits 150-fold selectivity for NTRS2 over NTRS1 [30].

SR 48692, 2-[[1-(7-chloroquinolin-4-yl)-5-(2,6-dimethoxyphenyl)-1H-pyrazol-3-carbonyl]amino] adamantane-2-carboxylic acid is a neurotensin antagonist selective for NTRS1 over NTRS2 20.

SR 142948, 2-[[5-(2,6-dimethoxyphenyl)-1-(4-(*N*-(3-dimethylaminopropyl)-*N*-methylcabamoyl)-2-isopropylphenyl)-1H-pyrazole-3-carbonyl]amino]adamantane-2-carboxylic acid is a synthetic NTR antagonist that completely inhibits [^125^I]ITyr^3^-NT specific binding to HT-29 cells [31].

To evaluate specific binding, 5 × 10^5^ HT-29 cells previously prepared according to the cell culture protocol were incubated with 70 nM of NTRC 824 (Bio-Techne Ltd., Abingdon, UK, dissolved in 0.1 M DMSO), 30 nM of SR 48692 (Bio-Techne Ltd., Abingdon, UK, dissolved in 0.1 M DMSO), and 10 nM of SR 142948 (Bio-Techne Ltd., Abingdon, UK, dissolved in 0.1 M DMSO) for 1.5 h. Afterwards, the medium containing unbound antagonists was removed and the cells were washed with 2 mL of fresh DMEM medium. After the baseline acquisition, 0.6 nmol of ^68^Ga-DOTA-NT was incubated and the uptake and cell retention were assessed according to the method described above.

### 2.7. Biodistribution Studies

All experiments were conducted according to the guidelines of EU Directive 2010/63 approved by The Romanian National Sanitary Veterinary and Food Safety Authority (NSVFSA, approval no. 442/25.02.2019).

Biodistribution studies of ^68^Ga-DOTA-NT and ^177^Lu-DOTA-NT were carried out on 8–10 weeks old, athymic nude male NU/J mice (Jackson Laboratory, Bar Harbor, ME, USA), weighting 26 ± 2.5 g.

The mice were inoculated 7 days prior to peptide administration with 5 × 10^6^ HT-29 colon cancer cells. All animals were anesthetized before testing by administration of a mixture of 7.5 mg/mL ketamine (Narkamon Bio, Bioveta Romania S.R.L., Cluj-Napoca, Romania), 1.5 mg/mL xylazine (Narcoxyl 2, MSD Animal Health, Kenilworth, NJ, USA) and 0.25 mg/mL acepromazine (Sedam, Pasteur Filiala Filipești S.A., Filipeștii de Pădure, Romania)

The radiopharmaceuticals were injected intravenously in the tail vein with 2.8 ± 0.4 Bq of ^68^Ga-DOTA-NT per animal or 9.7 ± 1.6 MBq of ^177^Lu-DOTA-NT, respectively. The actual injected activity took over in the bloodstream was corrected by subtraction of residual activity left in the syringe, and the activity remaining at the injection point (animal tail). Mice injected with ^68^Ga-DOTA-NT were sacrificed at 30- or 60-min post-injection (p.i.). Mice injected with ^177^Lu-DOTA-NT were divided into three groups, corresponding to different dissection time (60 min, 7 days, 14 days p.i.). The organs of interest were weighted on an analytical scale ALT 220-4M (Kern & Sohn GmbH, Balingen, Germany) and their activities were measured with calibrated multi-channel-analyser for γ-spectroscopy equipped with NaI (Tl) detector Mucha (Raytest GmbH, Straubenhardt, Germany). The biodistribution was calculated as percent of injected dose per gram of organ (% ID/g) ± standard deviation.

### 2.8. Statistical Analysis

The data were expressed as means ± standard deviation (*n* = 4). Comparison of results among the groups (%ID/g vs. tracers, %ID/g vs. tracers and organs, %ID/g vs. tracers and tumour cells), was carried out by one-way analysis of variance (ANOVA) and Tukey post-hoc analysis using SPSS v26 (IBM, New York, NY, USA). The results were considered significant for a *p*-value lower than 0.05.

## 3. Results

### 3.1. Labelling of DOTA-NT

There are two limitations in ^68^Ga use, namely its short half-life and the limited activity available from a generator (which is dependent on the initial activity of ^68^Ge loaded on the generator). Also, the maximum generated activity is continuously decreasing in time due to the decay of the loaded parent nuclide—^68^Ge. Therefore, each batch of ^68^Ga–labelled peptide was freshly prepared just before its use. These limitations compel to shorten the time required by synthesis and experiments, and also to maximize the overall yield of the preparation process.

The sole Ga isotope produced in the generator is ^68^Ga, produced from ^68^Ge by electronic capture, as carrier-free ^68^Ga. This is a huge advantage over the cyclotron route, which requires additional, time-consuming purifying steps and provides radiolabelled compounds with lower purity; the drawback consists of limited activity and the dead time between two batches (approx. 5–6 h), required by re-generation of sufficient quantity of ^68^Ga for the next synthesis.

**Figure 3:**
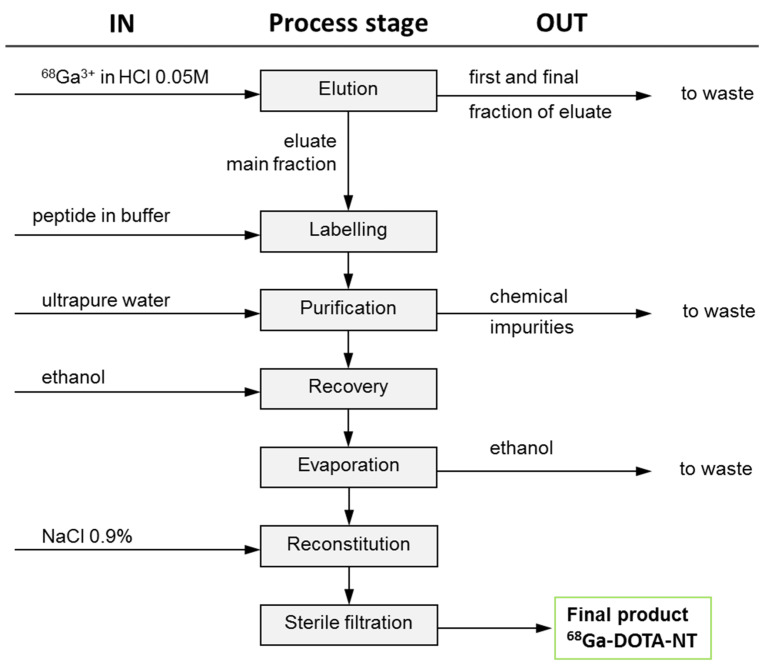
Flowchart of ^68^Ga-DOTA-NT production.

**Figure 4:**
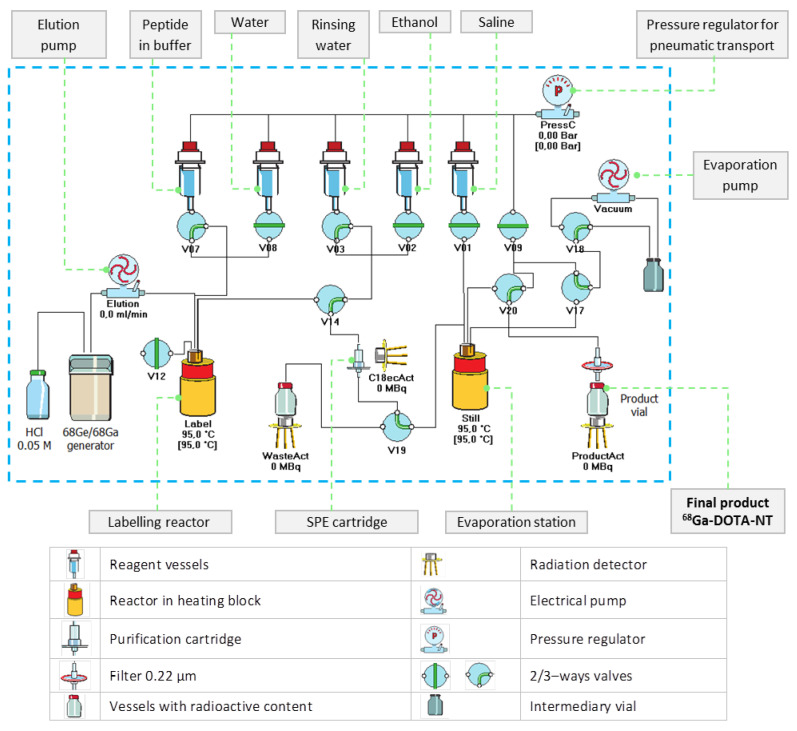
Graphical representation of ^68^Ga-DOTA-NT preparation process, edited from Absynth graphical interface (in blue frame); actual state is showing the system ready for labelling.

According to the manufacturer of the generator, a total volume of 4 mL HCl solution must be used for each synthesis. Only the most useful 2 mL of the eluate (the main fraction), concentrating the highest amount of ^68^Ga (extracted from the generator in form of ^68^GaCl_3_) should be used for labelling. The first and the last fractions of the eluate (a total of 2 mL—containing higher amount of zinc, produced during ^68^Ga decay inside the generator and HCl) are transferred to the waste. Compared with the tin oxide based generator, whose eluate usually contains small amounts of other impurities (cations of Ge, Fe, Sn, Zn) and should be purified prior to entering in contact with the peptides [32], this organic–column type generator does not require that step of purification. Moreover, this generator requires a much lower concentration of hydrochloric acid (0.05 M) compared to a tin oxide-based generator which typically requires a concentrations of 5 M and 10 M. To achieve high purity, short duration and high yield for ^68^Ga-labelling, preliminary experiments were carried out to investigate the process parameters (e.g., reaction temperature, pH, reaction time, evaporation time) that lead to optimal results. The optimal values of the automated process were implemented in a customized production script of proprietary Absynth controlling software. The synthesis flowchart and the scheme in the graphical interface of Absynth are presented in Figure 3 and Figure 4, respectively.

Preliminary tests revealed that for the labelling of neurotensin with ^68^Ga a more acidic pH is necessary (optimal 4.5) compared to the slightly higher pH in the case of ^177^Lu (pH = 5.5–6.0). In the case of ^68^Ga, the reaction time for total binding is shorter, 5 min at a temperature of 95 °C being sufficient to completely bind the radioisotope to the peptide. The labelling temperature was increased from 37 °C up to 95 °C to reduce the reaction time, without any detectable degradation of the radiolabelled peptide. The values of process parameters are presented in Table 1.

### 3.2. Radiochemical Purity and Stability

For avoiding background noise in imaging and harmful radioactive dose to the body after administration of radiopharmaceuticals, it is very important that the product does not undergo structural degradation over the shelf-life, as this may affect its pharmacokinetics and, therefore, the correctness of the effects it provides. The stability of a radiopharmaceutical depends on several factors such as: temperature, exposure to light, physical and chemical properties of the active substances, excipients, pharmaceutical form and properties of packaging materials. Radiochemical impurities may occur as a result of the degradation of the peptide due to radiolysis, temperature variations and by the action of oxidizing or reducing agents. Another possible cause can be the unstable trapping of the metallic ions by the chelator. In our studies, DOTA chelator exhibited good affinity both for Ga^3+^ and Lu^3+^, providing fast and stable binding (up to 4 h from EOS for ^68^Ga-DOTA-NT and 48 h from EOS for ^177^Lu-DOTA-NT).

**Figure 5:**
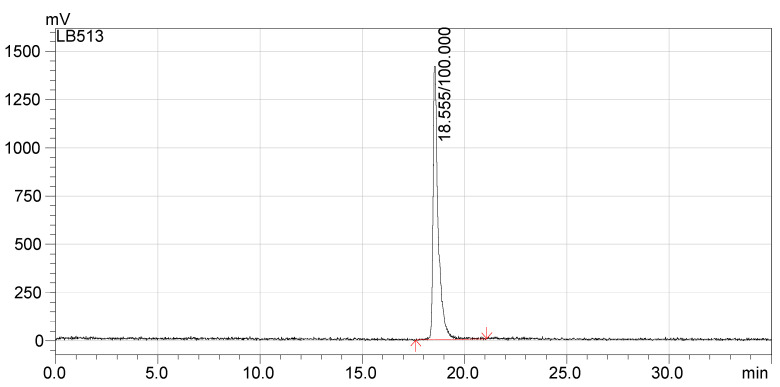
Radio-HPLC chromatogram of ^68^Ga-DOTA-NT.

**Figure 6:**
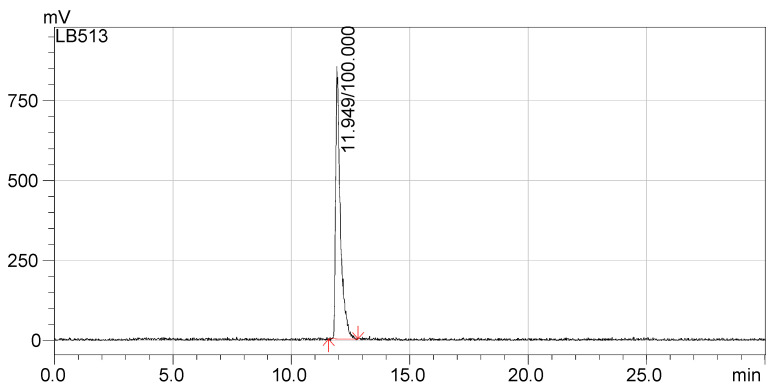
Radio-HPLC chromatogram of ^177^Lu-DOTA-NT.

The radio-HPLC chromatograms presented in Figure 5 and Figure 6 indicate that the synthesized compounds meet the radiochemical purity condition, with RCP > 99% for ^68^Ga-DOTA-NT (retention time 18.5 min) and >99% for ^177^Lu-DOTA-NT (retention time 11.9 min).

**Figure 7:**
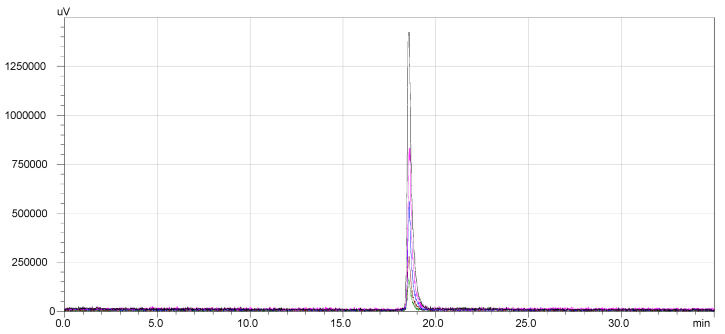
^68^Ga-DOTA-NT stability: overlapped chromatograms from EOS to EOS + 4 h.

The stability of ^68^Ga-DOTA-NT was assessed by radio-HPLC for 4 h after the end of labelling and purification process (EOS). Following the RCP analysis at the end of synthesis, other 4 samples were taken from the same vial at equal time intervals (1 h). The overlapped chromatograms are presented in Figure 7**.**

**Figure 8:**
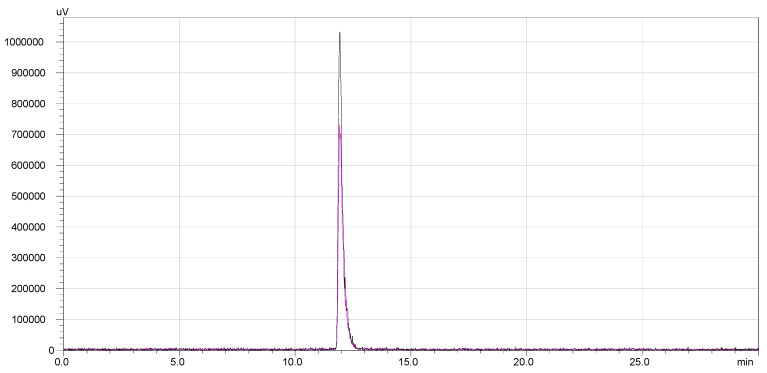
^177^Lu-DOTA-NT stability: overlapped chromatograms at EOS and EOS + 48 h.

The radiochemical stability of ^177^Lu-DOTA-NT was checked up to 48 h from the end of preparation. Both labelled compounds maintained their radiochemical purity throughout the investigation periods. No other peaks were identified in the subsequent chromatograms. Therefore, DOTA-NT did not undergo any structural modification and the radioisotopes were stably trapped by DOTA chelator. The overlapped chromatograms are presented in Figure 8.

### 3.3. In Vitro Uptake and Retention Measurement of ^68^Ga-DOTA-NT

**Figure 9:**
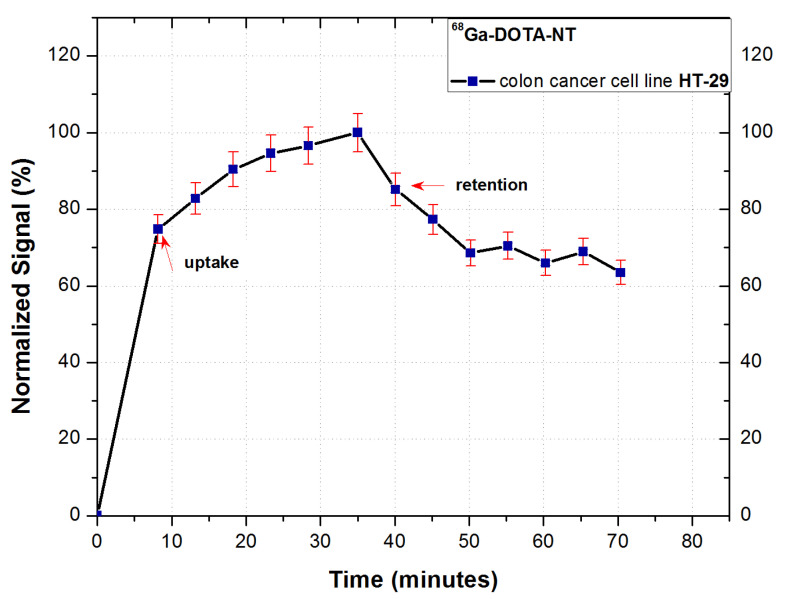
In vitro uptake/retention curve of ^68^Ga-DOTA-NT on HT-29 cancer cells. Data are presented as mean ± SD (*n* = 5).

The binding kinetics of ^68^Ga-DOTA-NT to NTR-expressing HT-29 cancer cells during incubation is presented in Figure 9.

Following the analysis of the cellular uptake of ^68^Ga-DOTA-NT, an exponential increase of the cell-associated activity can be observed, the trend of the uptake profile indicating a good dynamic of the tracer.

The highest cell retention was obtained for 0.6 nmol of NT, the maximum retention reaching 70% of the maximum measured activity during the uptake stage of analysis. This result confirms the expression of NT receptors on tumour cells membrane, as well as the ability of the peptide to form stable bonds with receptors. To verify the stability of binding cell, retention was studied for 35 min. A slight decrease in activity can be observed in the first 10 min of retention analysis (from 83% immediately after starting) followed by its stabilization around 70%.

### 3.4. In Vitro Specific Binding Assay

**Figure 10:**
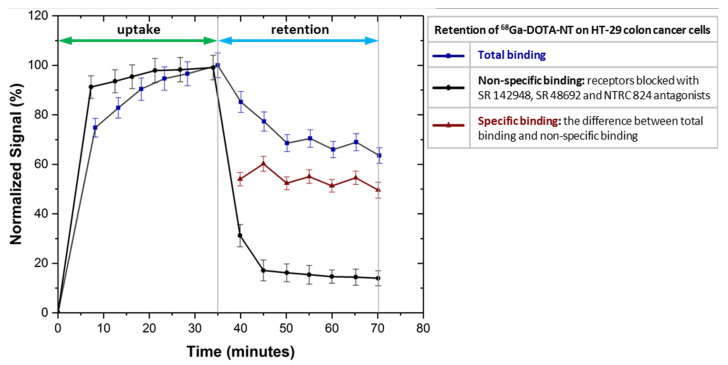
In vitro evaluation of specific binding of ^68^Ga-DOTA-NT on HT-29 cancer cells. Data are presented as mean ± SD (*n* = 5).

As can be seen in Figure 10, NTRC 824, SR 48692 and SR 142948 antagonists used to block NTRS1, NTRS2 and NTRS3 receptors inhibit the ^68^Ga-DOTA-NT binding to colon HT-29 tumour cells as the uptake profile trend is almost linear.

After removal of the radioactive medium the amount of non-specific binding of ^68^Ga-DOTA-NT to cells is about 18% compared to the value of total binding stabilized at 70%, normalized for the maximum detected value. Because all receptors are blocked, the amount of peptide retained by the cells is presumably due to other non-specific binding mechanisms such as: channel-forming mechanism, inverted micelle, barrel-stave pores, endocytosis mechanism etc. [33].

Subtracting the non-specific binding from the total binding we obtain a specific binding of about 52%. This result allows us to assume that applied in vivo ^68^Ga-DOTA-NT will be distributed mainly in tissues that express neurotensin receptors, the non-specific binding being too low to generate unwanted accumulations in other tissues.

### 3.5. In Vitro Binding Kinetics of ^177^Lu-DOTA-NT

**Figure 11:**
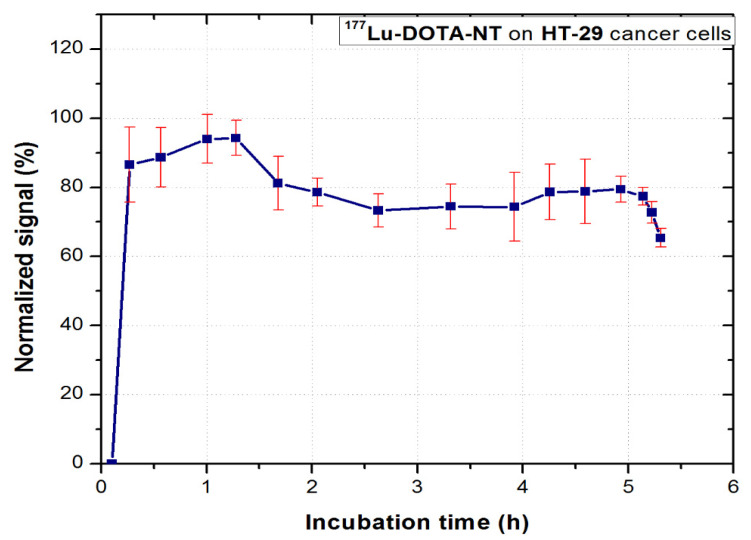
In vitro uptake/retention curve of ^177^Lu-DOTA-NT on HT-29 cancer cells. Data are presented as mean ± SD (*n* = 3).

^177^Lu-DOTA-NT was evaluated in vitro for 6–8 h to observe the binding kinetic profile of the compound to HT-29 tumour cells. The real-time dynamic measurement is shown in Figure 11.

In the first 90 min of incubation, the compound follows a trend of association with NT receptors. After removing the excess free compound in the medium, the cell dynamics was continuously measured. Stable retention on the cells (approx. 70% of the maximum association activity for 3.5 h) can be observed. This value is consistent with that obtained for ^68^Ga-DOTA-NT, which indicates that the ^68^Ga and ^177^Lu radioisotopes do not interfere in the process of peptide binding to its receptors.

After approx. 5 h from the incubation of the tracer, a sudden decrease of activity can be observed, which coincides with the second phase, critical for the therapy tracers, respectively the dissociation phase. At this stage, the tumour cells detach from the Petri dish due to the processes of apoptosis. They float in the culture medium and at subsequent rotations of the support they are no longer detected in the region they were initially attached. This method of analysis allows real-time visualization of these processes which confirms the possibility of using DOTA-NT as a theranostic agent. Since the stability of ^177^Lu-DOTA-NT has been assessed for up to 48 h post-synthesis with no degradation observed, the possibility of peptide degradation or release of radionuclide in the medium can be excluded.

### 3.6. Biodistribution

#### 3.6.1. ^68^Ga-DOTA-NT

**Figure 12:**
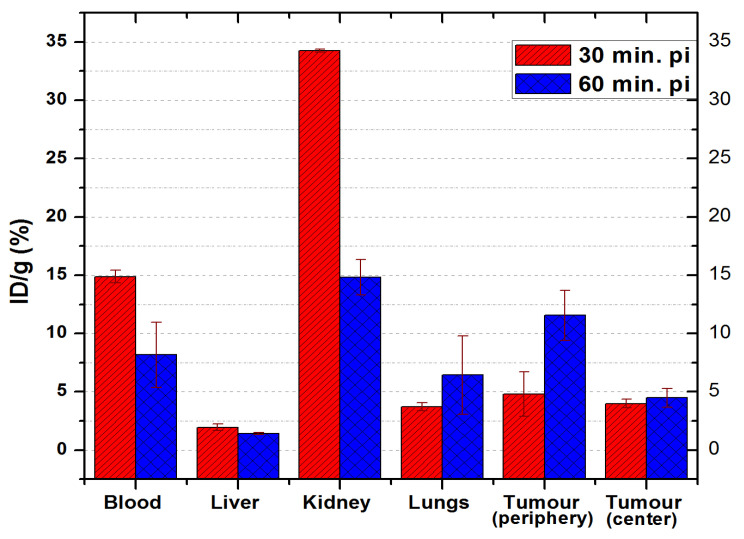
Biodistribution of ^68^Ga-DOTA-NT in NU/J mice. Data are presented as mean ± SD (*n* = 4).

The results of the biodistribution studies of ^68^Ga-DOTA-NT are summarized in Figure 12.

Significant uptake of ^68^Ga-DOTA-NT was observed in kidneys at 30 min p.i (34.26 ± 0.14%ID/g). It can be observed that 60 min p.i., the amount of tracer in the kidneys halves down to 14.82 ± 1.51% ID/g, most likely due to excretion through the renal system. No significant retention was observed in the liver (1.95 ± 0.28% ID/g at 30 min p.i. and 1.42 ± 0.073% ID/g at 60 min p.i.), suggesting that ^68^Ga-DOTA-NT was fairly water-soluble and does not associate into larger polypeptides chains, according to other studies [34,35].

The second most significant measured activity was detected in the blood, 14.87 ± 0.55% ID/g at 30 min p.i. respectively 8.17 ± 2.79% ID/g at 60 min p.i. This result, along with the observed decrease of the kidneys’ activity after 60 min, indicates a fast clearance of neurotensin.

The lungs and the tumour are the only organs in which a higher activity (accumulation) was registered at 60 min p.i. compared to the activity measured at 30 min p.i. Thus, in the lungs the activity increases from 3.71 ± 0.34% ID/g at 30 min p.i., up to 6.44 ± 3.36% ID/g at 60 min p.i.

Tumour vasculature is a critical micro-environmental factor associated with tumour response to the therapy and heterogeneous in both time- and location- dependent manner. Thus, for an accurate assessment, the tumour was sectioned into two parts: the centre of the tumour and the periphery. At 30 min p.i. the distribution of the tracer (activity) throughout the tumour mass is homogeneous, as the centre and the periphery of the tumour have close values: 4 ± 0.38% ID/g and 4.78 ± 1.91% ID/g respectively. At 60 min p.i. the activity at the tumour edge increases to 11.56 ± 2.14% ID/g compared to 4.5 ± 0.8% ID/g in the centre. This result highlights better vessel function and higher microvascular density (MVD) of the tumour periphery, than the tumour core.

#### 3.6.2. ^177^Lu-DOTA-NT

**Figure 13:**
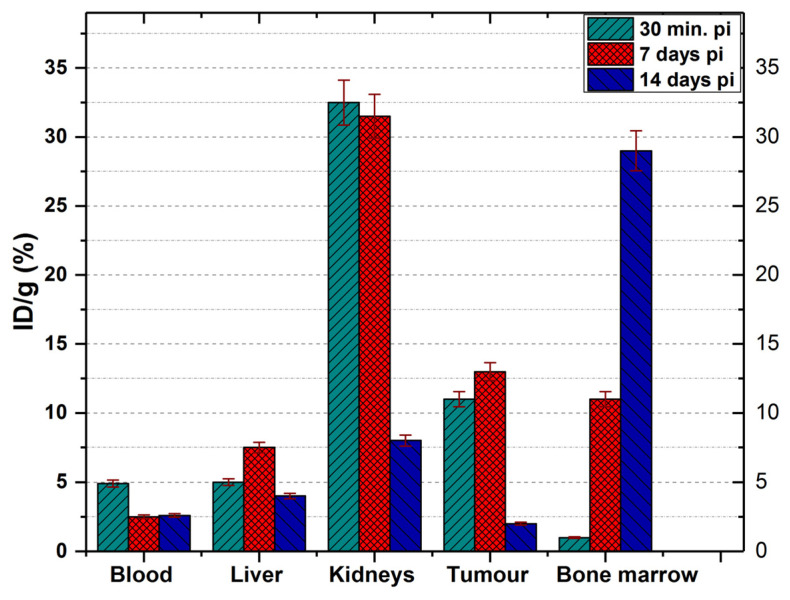
Biodistribution of ^177^Lu-DOTA-NT in NU/J mice. Data are presented as mean ± SD (*n* = 4).

**Figure 14:**
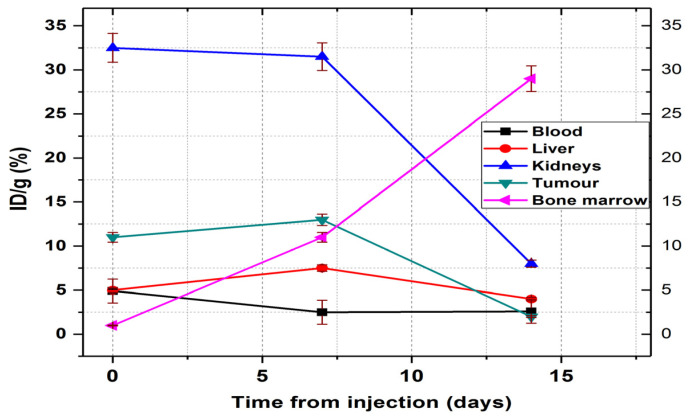
Biodistribution of ^177^Lu-DOTA-NT in NU/J mice. Data are presented as mean ± SD (*n* = 4).

The biodistribution of ^177^Lu-DOTA-NT was studied at 3 different times in order to observe the evolution of the tumour under the action of β^-^ radiation, but also how this compound can affect other organs by accumulation on them. The results are presented in Figure 13 and Figure 14.

At 30 min p.i. the tracer was found mainly in the kidneys (32.5 ± 1.6% ID/g), tumour (11 ± 0.6% ID/g), blood (4.9 ± 0.2% ID/g) and liver (5.1 ± 0.21% ID/g). At 7 days p.i., the amount of tracer in the blood decreases significantly (reaching 2.5 ± 0.15% ID/g) but increased the accumulation in the liver (7.5 ± 0.4% ID/g). At this time, the amount of ^177^Lu-DOTA-NT in the kidneys showed a slight decrease to 31.5 ± 1.2% ID/g.

Recent studies showed radiologic signs of liver steatosis during the treatment with ^177^Lu-DOTA-TATE, but an improvement by the end of the treatment period [36]. These findings, correlated with increased liver radioactivity at 7 days p.i., indicate a possibility that ^177^Lu-DOTA-NT may have a hepatotoxicity effect, but further evaluation is needed.

At 14 days p.i., the activity in the blood is kept at 2.6 ± 0.23% ID/g (similar to that reported at 7 days p.i.), and in the liver decreases significantly compared to that at 7 days p.i., reaching 4.3 ± 0.98% ID/g.

In the kidneys, the activity at 14 days decreases down to 8.1 ± 1.2% ID/g, a proportional decrease to that of the tumour (1.99 ± 0.35% ID/g).

The only organ in which activity growth is maintained 14 days after injection is the bone marrow, where it reaches 29.45 ± 2.1% ID/g. Comparing the accumulated activity in the bone marrow with the accumulated radioactivity of the radiopharmaceutical in the blood, we could easily observe in Figure 14 that the activity measured in the femur marrow increases much more than the decrease in the blood. This dynamic process indicates a very rapid absorption of the ^177^Lu–radiolabelled peptide in the bone marrow.

## 4. Discussion

Regulatory peptides, such as neurotensin, are suitable agents for specific delivery of radioisotopes to tumour cells, for both diagnostic and therapeutic applications [37,38]. The aim of this study was the investigation of neurotensin as a potential agent used in diagnosis and therapy of colon cancer when coupled with ^68^Ga and ^177^Lu, respectively.

In vitro evaluation of the binding kinetics (uptake and retention) on HT-29 colon cancer cells revealed a maximum of 70% retained activity from the maximum recorded during uptake. This result confirms the expression of NT receptors on tumour cells membrane, as well as the ability of the peptide to form stable bonds with receptors. Based on the results obtained for the specific binding assay, we were able to further determine that the non-specific binding represents 18% of the total binding, and the remaining 52% is attributed to the specific binding of neurotensin to its receptors (NTRS1, NTRS2 and NTRS3), In the case of ^177^Lu-DOTA-NT, 5 h after cell incubation, the cell retention profile follows a sudden decreasing trend of radioactivity, which might signify the colon cancer cells death and detachment from the Petri dish. These results demonstrate the need for additional, more precise investigation of ^177^Lu-DOTA-NT cytotoxic effects.

In vivo biodistribution studies in athymic nude mice bearing colon tumours confirmed the increased potential of ^68^Ga-DOTA-NT to target this type of cells, with 4.78 ± 1.91% ID/g at the tumour periphery.

Although previous studies showed that neurotensin has a low stability in blood, our results indicate that the use of ^68^Ga-radiolabelled peptide may be feasible for imaging of tumour from 30 min up to 1 h p.i. This time interval seems to sufficiently allow the distribution and accumulation of radiolabelled peptide into the body. A longer time p.i. is not feasible, as long as it leads to losing too much activity due to the short half-life of ^68^Ga.

A high uptake (6.44 ± 3.36% ID/g at 60 min p.i) was measured in the lungs. This result can be explained by the previous discoveries that pulmonary parenchyma is an important site of neurotensin metabolism [39] and a high density of NTRS1 is expressed by lung pleura [40]. No uptake of ^68^Ga-DOTA-NT was detected in the brain, most certainly due to low blood brain barrier (BBB) penetration of neurotensin, otherwise a non–BBB–penetrant neuropeptide.

In the case of ^177^Lu-DOTA-NT, at 30 min p.i, the highest amount of injected dose is found in the kidneys. This level is maintained at 7 days p.i., but at 14 days p.i. it decreases to 8.1 ± 1.2% ID/g. From previously reported data, treatment with ^177^Lu administered in the form of ^177^Lu-DOTA-TATE has been shown to cause radiation-induced renal damage [41]. To reduce or avoid this side effect, different molecules (like Human Antioxidation Protein α_1_-Microglobulin) have been developed that can effectively limit this effect. To assess whether the residual activity of ^177^Lu-DOTA-NT after 14 days p.i. causes nephrotoxicity, future studies are needed.

The challenge associated with the use of small peptides is the rapid in vivo degradation by peptidases that might require their activity inhibition [42], before peptide–based drug administration. An interesting finding was that, even without inhibiting the proteolytic enzymes, the activity continued to accumulate in the tumours for up to 7 days and then decreased. The possible causes of decreasing may be: the peptide instability (although in this case the peptide was stable for more than a few hours), or the tumour cells apoptosis, which is a desirable effect of the ^177^Lu. However, further investigations and an additional study in conditions of inhibited degrading enzymes are needed.

Despite these promising results, a cause for concern is the constant accumulation in the bone marrow, increasing from 0.98 ± 0.32% ID/g at 30 min p.i. to 29.45 ± 2.1% ID/g at 7 days p.i. Future toxicity studies will be performed to evaluate long-term bone marrow toxicity, whether can cause myelosuppression, and also to optimize the dose at which the treatment with ^177^Lu-DOTA-NT is safe for administration.

## 5. Conclusions

The results obtained using NT coupled with ^68^Ga for the detection of colon cancer and the potential of this peptide to be used in therapy coupled with ^177^Lu are arguments to extend the studies of this peptide as a potential theranostic agent, with and without having the peptidases previously inhibited. Increased in vitro uptake and retention in colon HT-29 cancer cells have been proved. Also, in vivo biodistribution results have proved the ability of NT to preferentially target the tumour site. Further, additional studies are necessary to assess if there is a long-term toxic effect due to ^177^Lu-DOTA-NT accumulation in the kidneys, liver or bone marrow.

The presented synthesis methods of ^68^Ga- and ^177^Lu-DOTA-NT are reproducible, producing radiopharmaceuticals compliant with the requirements for radiochemical purity according to current pharmaceutical regulations (European Pharmacopoeia) and which are stable throughout the entire shelf-life. DOTA chelator exhibited good affinity both for Ga^3+^ and Lu^3+^, providing fast and stable binding. The preparation processes of ^68^Ga-DOTA-NT and ^177^Lu-DOTA-NT were established in terms of pH, reaction and evaporation times and temperatures, to obtain a high labelling yield within a short overall preparation time. In the case of ^68^Ga, the successful automation of the entire process including elution, labelling and purification allowed to obtain fully reproducible results. This also virtually eliminates the operators’ exposure during synthesis and also helps to avoid human errors.

## Figures and Tables

**Table 1:** Labelling parameters for ^68^Ga- DOTA-NT and ^177^Lu-DOTA-NT.

Parameter	^68^Ga-DOTA-NT	^177^Lu-DOTA-NT
Peptide amount	24 nmol	
Buffer	Ammonium acetate (1M)	
Labelling pH	4.5–4.8	5.5–6.0
Reaction volume	<2.5 mL	
Reaction time	5 min	10 min + 10 min
Temperature	95 °C	100 °C + natural cooling
Ethanol amount	1 mL	—
Evaporation temperature	95 °C	—
Evaporation time	360 s	—
Overall preparation duration	22 min	approx. 30 min
Radiochemical purity ^1^	> 99%	> 99%
Stability	> 4 h	> 48 h

^1^ assessed by radio-HPLC.

## Data Availability

The data of this study are available within this article, further inquiries may be directed to the authors.
